# Molecular detection of *Mycoplasma synoviae* and avian reovirus infection in arthritis and tenosynovitis lesions of broiler and breeder chickens in Santa Catarina State, Brazil

**DOI:** 10.4102/jsava.v90i0.1970

**Published:** 2019-11-29

**Authors:** Carolina Reck, Álvaro Menin, Mariana F. Canever, Celso Pilati, Luiz C. Miletti

**Affiliations:** 1 Department of Animal Production, Center for Agricultural Sciences, Santa Catarina State University, Lages, Brazil; 2 VERTÀ, Research and Veterinary Diagnostic Institute, Curitibanos, Santa Catarina, Brazil; 3 Department of Biosciences and One Health, Federal University of Santa Catarina, Curitibanos, Santa Catarina, Brazil; 4 Department of Animal Production, Santa Catarina State University, Center for Agricultural Sciences, Lages, Santa Catarina, Brazil; 5 Department of Veterinary Medicine, Center for Agricultural Sciences, Santa Catarina State University, Lages, Santa Catarina, Brazil

**Keywords:** poultry, arthritis, *Mycoplasma synoviae*, avian reovirus, PCR, polymerase chain reaction

## Abstract

Infectious arthritis or tenosynovitis in broiler and breeder chickens results in major loss of productivity because of reduced growth and downgrading at processing plants. The most common causative agents of avian infectious arthritis are the bacterium *Mycoplasma synoviae* and avian reoviruses (ARVs) (family Reoviridae, genus *Orthoreovirus*). In this study, we evaluated the occurrence of these two pathogens in arthritis or tenosynovitis lesions of broilers and breeder flocks in southern Brazil using molecular detection. Tissue sections from tibiotarsal joints with visible lesions from 719 broilers and 505 breeders were analysed using pathogen-specific polymerase chain reaction (PCR) assays. In breeders, 41.2% (*n* = 296) of lesions were positive for *M. synoviae*, 26.4% (*n* = 190) were positive for ARV, while co-infection was present in 12.2% (*n* = 88) of the samples. In broilers, 20.8% (*n* = 105) of lesions were positive for *M. synoviae*, 11.9% (*n* = 60) for ARV and 7.7% (*n* = 39) of these cases were positive for both pathogens. Post-mortem examination revealed lesions with varying degrees of gross pathological severity. Histopathological examination showed intense, diffuse lymphohistiocytic inflammatory infiltrates with heterophil accumulation, primarily in the synovial capsule and digital flexor tendon, in all samples. Improved strategies for early detection and control of these major avian pathogens are highly desirable for preventing the spread of infection and reducing economic losses in the poultry industry.

## Introduction

The poultry industry is the largest component of the meat industry in Brazil and shows a steady increase in the annual growth (Nääs et al. 2015). Brazil has been the world’s major chicken meat exporter (to > 150 countries) since 2004 (Campos 2016). For the poultry industry worldwide, infectious arthritis or tenosynovitis in broilers and breeders caused by the bacterium *Mycoplasma synoviae* (division Firmicutes) and avian reoviruses (ARVs) (family Reoviridae, genus *Orthoreovirus*) is a serious economic and health problem. These infections result in major economic losses due to reduced production and downgrading of meat at processing plants (Moreira, Cardoso & Coelho 2017), and the associated pain and limitation of movement impact negatively on animal welfare.

A better understanding of the dynamics of infection within a flock is essential for reducing such losses and for developing more effective surveillance and control strategies for these two major avian pathogens (Nham et al. 2017; Sun et al. 2017).

*Mycoplasma synoviae* causes arthritis, synovitis, respiratory diseases, increased mortality and immunosuppression, as well as reduced egg production and hatchability in chickens and turkeys (Landman & Feberwee 2012; Lockaby et al. 1998). Avian reoviruses (ARVs), a type of ribonucleic acid (RNA) virus, are associated with severe arthritis or tenosynovitis, chronic respiratory diseases, leg weakness, immunosuppression and malabsorption syndrome (Landman & Feberwee 2012; Zhong et al. 2016). Mixed infection caused by both *M. synoviae* and ARV in chickens results in exacerbation of clinicopathological effects (Dobson & Glisson 1992; Moreira et al. 2017; Reck et al. 2012).

Techniques typically used for routine diagnostics, including serological analysis (e.g. enzyme-linked immunosorbent assay) and isolation methods, are laborious and time-consuming. In addition, the serological method only gives a history of infection, which results in the delay of treatment resulting in the further spread of infection, and analysis is often unreliable because of non-specific reactions and reagent cross-reactivity (Feberwee et al. 2005). For these reasons, improved techniques for rapid, early detection of *M. synoviae* and ARV are highly desirable for preventing the spread of infection and reducing economic losses in the poultry industry.

We describe here, for the first time, systematic evaluation of the occurrence of *M. synoviae* and ARV infection in arthritis or tenosynovitis lesions in broiler and breeder flocks in southern Brazil.

## Material and methods

The study population comprised of 1224 chickens collected from farms in the Santa Catarina state (southern Brazil) in 2015. By 2017: 719 broilers (33 flocks) (age 6–7 weeks) and 505 breeders (19 flocks) (age 66–67 weeks) were found with visible arthritic lesions in tibiotarsal joints. Tissue samples from the affected joints, including the synovial membrane and digital flexor tendon, were collected in processing plants, and preserved in liquid nitrogen for nucleic acid extraction and formalin-fixed for histopathological examination. Legs containing the affected joints were also examined. The chi-square test was used for statistical analysis.

Deoxyribonucleic acid (DNA) from tissue samples were extracted as described previously (Triant & Whitehead 2009). For genomic RNA extraction from avian reovirus (ARV), Trizol (Invitrogen, Carlsbad, CA, United States [US]) was used according to the manufacturer’s instructions. Reverse-transcriptase-Polymerase chain reaction (RT-PCR) for first-strand complementary DNA synthesis from viral RNA was performed using a Protoscript M-MuLV First-Strand cDNA (complementary deoxyribonucleic acid) Synthesis kit (New England Biolabs, Ipswich, MA, US) as per the manufacturer’s protocol. Deoxyribonucleic acid and cDNA were used as a template for polymerase chain reaction (PCR) reaction.

Detection of *M. synoviae* and ARV by PCR was performed as described by Reck et al. (2013). The multiplex PCR (mPCR) reactions were performed in a 20 *µ*L volume. Extracted DNA and synthesised cDNA were mixed in equal proportions of each 100 ng, thereby providing the template for *M. synoviae* and ARV detection, respectively. The mPCR reactions were carried out using optimised MgCl_2_, 10× PCR buffer II (Invitrogen, Carlsbad, CA, US), 200 mM of each deoxynucleotide (dNTP) (Invitrogen, Carlsbad, CA, US), 20 pmol of each primer and 5 units of *Taq* DNA polymerase (Invitrogen, Carlsbad, US). The cycling protocol consisted of an initial denaturation at 94 °C for 5 minutes, followed by 35 cycles of denaturation at 94 °C for 1 min, annealing 52.6° for 1 min and extension was at 72 °C for 1 min. The sample was heated at 72 °C for 10 min for a final extension. Each mPCR run included positive and negative controls for PCR reagents and sample extractions. The negative control did not contain template DNA or cDNA and consisted of PCR mastermix, all four sets of primers and deionised water. The mPCR amplification products were analysed by means of electrophoresis in 1.5% (weight per volume [w/v]) agarose gel and TBE buffer. Polymerase chain reaction was performed with sets of oligonucleotide primers that specifically amplify the target sequence of the S1 gene (532 base pairs [bp]) from the ARV (Xie et al. 1997) and MS-16S rRNA sequence (207 bp) from *M. synoviae* (Lauerman et al. 1993).

Tissue samples were fixed in 10% neutral buffered formalin, dehydrated by an ethanol solution gradient, paraffin-embedded and cut into 4 *µ*m sections. Serial sections were haematoxylin and eosin stained, examined by an Olympus BX-40 microscope, and images were digitally recorded.

### Ethical considerations

All animal procedures were previously evaluated and approved by the Ethics Committee on Animal Experiments (CEUA/CAV/UDESC), Protocol no. 1.31.11.

## Results

**FIGURE 1:**
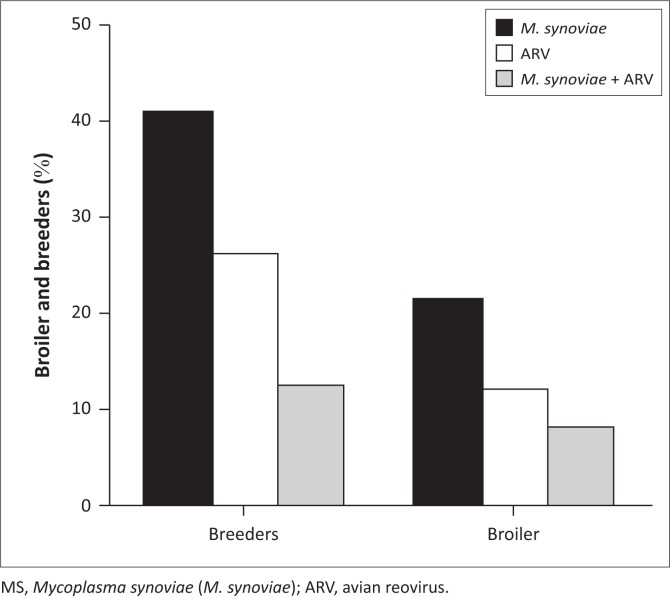
Polymerase chain reaction analysis of *M. synoviae* and avian reovirus detection in 1224 samples (719 broilers, 505 breeders) from 41 and 17 flocks, respectively. Results show the percent of samples positive for *M. synov*iae, avian reovirus and simultaneous *M. synoviae* and avian reovirus infection (MS+ARV).

Polymerase chain reaction analysis of 719 arthritic lesions from breeders showed positive amplification for *M. synoviae* and ARV in 41.2% (*n* = 296) and 26.4% (*n* = 190) of samples, respectively (Figure 1). Further, 12.2% (*n* = 88) of samples were positive and 20.2% (*n* = 145) were negative for both pathogens. Analysing 505 arthritic lesions from broilers, 20.8% (*n* = 105) were positive for *M. synoviae*, 11.9% (*n* = 60) for ARV and 7.7% (*n* = 39) for both pathogens. Meanwhile 59.6% (*n* = 301) of samples were negative for both pathogens (Figure 1).

**FIGURE 2:**
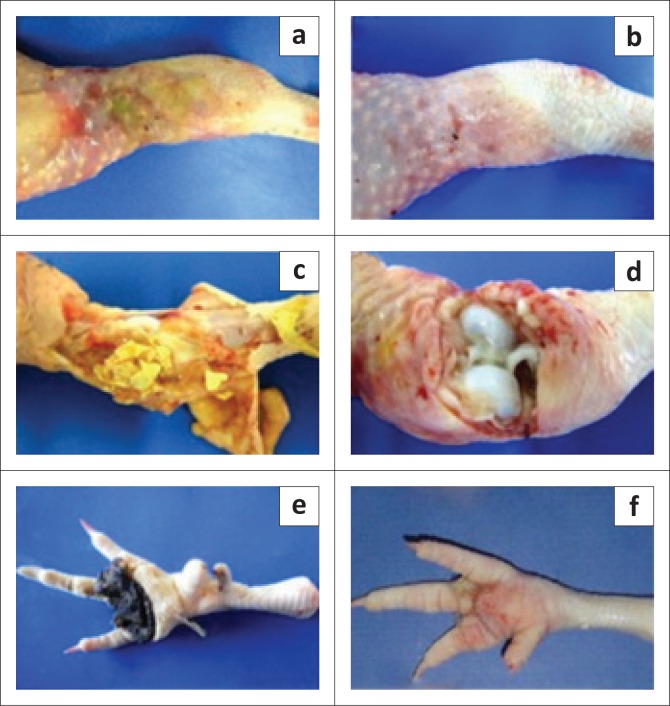
Gross arthritic lesions in tibiotarsal joint and pododermatitis, in broilers and breeders infected by *M. synoviae* and/or avian reovirus. (a, b) Severe gross lesions of infectious arthritis. Note swelling of joints. Reddish-purple (a) and green areas (b) indicate haemorrhagic lesions. (c) Tibiotarsal joint of broiler with increased synovial fluid and petechiae in synovial membrane. (d) Presence in breeder of caseous yellowish exudate into tibiotarsal joint and surrounding tissue. (e, f) Pododermatitis with the presence of mild lesions (in broiler) (e), and severe pathology with plantar abscess formation (in breeder) (f).

Post-mortem examination in all chickens (719 breeders, 505 broilers) revealed variable degrees of footpad dermatitis (pododermatitis) and tibiotarsal joint arthritis. Gross lesions included caseous exudate filling the joint cavity, increased synovial fluid and petechiae in synovial membranes (Figure 2a–f).

Intra-articular purulent exudate was observed in 39.1% (*n* = 281) of breeders (Figure 2c) and 21.4% (*n* = 108) of broilers (Figure 2d). Pododermatitis of varying degrees of severity was observed in all breeders and 37.6% (*n* = 190) of broilers (Figure 2e–f).

**FIGURE 3:**
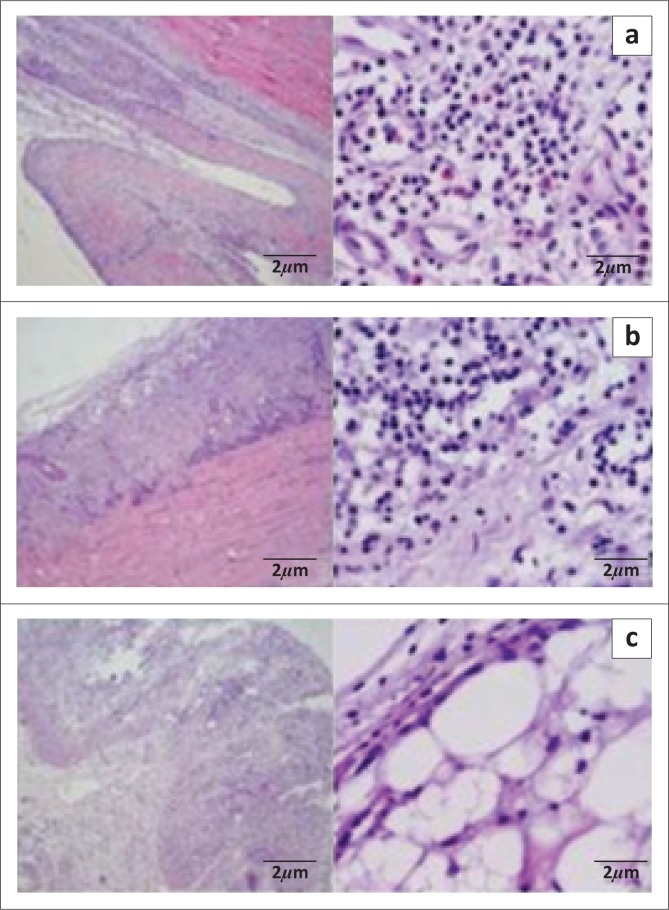
Histopathological lesions in the tibiotarsal arthritis in broilers and breeders infected by *M. synoviae* and/or avian reovirus. Formalin-fixed, paraffin-embedded arthritic articulation tissue sections from broilers and breeders were stained with haematoxylin and eosin. Slides shown correspond to the typical findings from broilers and breeder chickens. (a) Intense and diffuse lymphohistiocytic inflammatory infiltrate with accumulation of heterophils primarily in the synovial capsule. (b) Hyperplasia and hypertrophy of synovial cells with formation of villi and lymphohistiocytic infiltrate with heterophils accumulation in the synovial spaces. (c) Cartilage general matrix destructuring in different degrees and presence of inflammatory infiltrate. Left panels, slides shown at 10× magnification; scale bar = 200 *µ*m. Right panels, slides shown at 40× magnification; scale bar = 20 *µ*m.

Histopathological analysis of arthritic lesions of tibiotarsal joints revealed intense, diffuse lymphohistiocytic inflammation with heterophil accumulations, primarily affecting the synovial capsule and digital flexor tendon, in all samples (Figure 3a). In many cases, we also observed hyperplasia and hypertrophy of synovial cells with the formation of villi and/or necrosis to varying degrees (Figure 3b–c).

In this study, we observed a high occurrence of *M. synoviae* and ARV, alone or in combination, in samples of the arthritic lesions of breeders and broilers from farms in southern Brazil (Figure 1). It is likely that *M. synoviae* frequency is often underestimated because of the widespread use of antibiotic treatments in breeder flocks and the short lifespan of broilers (Landman et al. 2008). Transmission of ARV between flocks is variable, because young chickens are sometimes more susceptible to viral infection, and transmission in adult chickens is facilitated by pododermatitis lesions (Jones & Georgiou 1984).

We observed a high frequency of *M. synoviae* infection in tibiotarsal arthritic lesions of breeders (Figure 1), although they were progeny from *M. synoviae*-negative breeder flocks, suggesting that infection occurred after hatching. This pathogen spreads quickly among animals in naturally *M. synoviae*-infected breeder flocks, resulting in severe economic losses (Marois et al. 2005). In our samples as well, the RT-PCR analysis revealed a high frequency of ARV (Figure 1).

In Brazil, infectious arthritic lesions in broilers are the primary reason for the partial downgrading of carcasses at processing plants (Giotto et al. 2008). Our molecular analyses (PCR and RT-PCR) of arthritic lesions showed a high frequency of positive samples for *M. synoviae* and ARV and co-infection for both pathogens. In these cases, efficient control measures would involve rapid slaughter of flocks positive for *M. synoviae* or ARV, control of vertical transmission and measures of biosecurity on farms (Feberwee, De Vries & Landman 2008; Landman et al. 2008).

Co-infection with *M. synoviae* and ARV is often observed in turkey and chicken flocks, and may be associated with severe cases of arthritis or tenosynovitis and systemic syndromes, including chronic respiratory disease and decreased growth (Landman & Feberwee 2012; Reck et al. 2012). We observed mixed infection from arthritic lesions in both broilers (7.7%) and breeders (12.2%) (Figure 1). Cell-mediated immune responses play crucial roles in the control of both *M. synoviae* and ARV infections and are involved in immunopathologic responses associated with the formation of arthritic lesions (Reck et al. 2012; Senties-Cue, Shivaprasad & Chin 2005). Mixed infection by *M. synoviae* and ARV in chickens results in exacerbation of clinicopathological effects (Moreira et al. 2017; Reck et al. 2012), suggesting a possible synergistic interaction of these two pathogens in immunosuppression (Ni & Kemp 1995; Senties-Cue et al. 2005). In the present study, cases of ARV infection occurred mainly as part of mixed infection with *M. synoviae* in both broilers and breeders.

Histopathological investigations confirmed the presence of infectious arthritis (Figure 3). Additionally, observations, particularly, the diffuse lymphohistiocytic infiltrate with heterophil accumulation in the synovial capsule and digital flexor tendon of samples, that are PCR-positive for *M. synoviae* and ARV, support the diagnosis of infectious arthritis caused by both avian pathogens (Bradbury & Garuti 1978; Ni & Kemp 1995; Reck et al. 2012).

Of our total sample of arthritic lesions (719 breeders, 505 broilers), 20.2% (*n* = 145) and 59.6% (*n* = 301) of samples from breeders and broilers, respectively, were negative for both *M. synoviae* and ARV by PCR. Such cases may have involved other pathogens, e.g., *Escherichia coli* or *Staphylococcus aureus* (Coura et al. 2017; Jungherr 1959; Kibenge, Robertson & Wilcox 1982). In this study, we isolated potential pathogens, such as *Escherichia coli, Pseudomonas aeruginosa, Staphylococcus aureus, Staphylococcus intermedius, Enterococcus cloacae, Aeromonas* sp., *Klebsiella* sp., *Pasteurella* sp., *Streptococcus* sp. and *Candida* sp. (data not shown). In addition, *M. synoviae* and ARV are known to cause chronic progressive disease, and tissue damage may be associated with disease immunopathology, which are negative for both pathogens in PCR assay.

## Conclusion

The findings presented here clearly indicate high frequencies of single and mixed infection by *M. synoviae* and ARV in tibiotarsal arthritic lesions in broilers and breeders in southern Brazil.

## References

[CIT0001] Bradbury J.M. & Garuti Y.A., 1978, ‘Dual infection with *Mycoplasma synoviae* and a tenosynovitis-inducing reovirus in chickens’, *Avian Pathology* 7(3), 407–419. 10.1080/03079457808418294 18770394

[CIT0002] Campos A., 2016, ‘Brazil’s poultry industry’, *Monitor* 2, 3–18, viewed 12 February 2019, from https://reporterbrasil.org.br/wp-content/uploads/2016/09/Indu%CC%81stria-do-Frango-ING-WEB.pdf.

[CIT0003] Coura F.M., Diniz S.A., Silva M.X., Arcebismo T.L.M., Minharro S., Feitosa A.C.F. et al., 2017, ‘Phylogenetic group of *Escherichia coli* isolates from broilers in Brazilian poultry slaughterhouse’, *Scientific World Journal* 2017, Art ID 5898701, 7 pages. 10.1155/2017/5898701 PMC5654288 29130064

[CIT0004] Dobson K.N. & Glisson J.R., 1992, ‘Economic impact of a documented case of *Orthoreovirus aviario* infection in broiler breeders’, *Avian Diseases* 36(3), 788–791. 10.2307/1591786 1329715

[CIT0005] Feberwee A., De Vries T.S. & Landman W.J., 2008, ‘Seroprevalence of *Mycoplasma synoviae* in Dutch commercial poultry farms’, *Avian Pathology* 37(6), 629–633. 10.1080/03079450802484987 19023760

[CIT0006] Feberwee A., Mekkes D.R., De Wit J.J., Hartman E.G. & Pijpers A., 2005, ‘Comparison of culture, PCR, and different serologic tests for detection of *Mycoplasma gallisepticum* and *Mycoplasma synoviae* infections’, *Avian Diseases* 49(2), 260–268. 10.1637/7274-090804R 16094832

[CIT0007] Giotto D.B., Zimermann C.F., Cesco M.A.O., Borges Fortes F.B., Pinheiro D., Hiller C.C. et al., 2008, ‘Impacto econômico de condenações post mortem de frangos de corte em um matadouro-frigorífico na região sul do Brasil’, Proceedings of the 35 Congresso Brasileiro de Medicina Veterinária, Gramado, Brazil, 19–22 October.

[CIT0008] Jones R.C. & Georgiou K., 1984, ‘*Reovirus-*induced tenosynovitis in chickens: The influence of age at infection’, *Avian Pathology* 13(3), 441–457. 10.1080/03079458408418546 18766859

[CIT0009] Jungherr E., 1959, ‘Bacterial arthritis and tenosynovitis in poultry’, *Laboratory Investigation* 8, Nov-Dez 1376–1383. 14408210

[CIT0010] Kibenge F.S., Robertson M.D. & Wilcox G.E., 1982, ‘*Staphylococcus aureus* isolated from poultry in Australia. II. Epidemiology of strains associated with tenosynovitis’, *Veterinary Microbiology* 7(5), 485–491. 10.1016/0378-1135(82)90065-7 7164340

[CIT0011] Landman W.J. & Feberwee A., 2012, ‘Longitudinal field study on the occurrence of *Mycoplasma synoviae* in Dutch turkey flocks with lameness and experimental induction of the condition’, *Avian Pathology* 41(2), 141–149. 10.1080/03079457.2011.652595 22515533

[CIT0012] Landman W.J., Mevius D.J., Veldman K.T. & Feberwee A., 2008, ‘*In vitro* antibiotic susceptibility of Dutch *Mycoplasma synoviae* field isolates originating from joint lesions and the respiratory tract of commercial poultry’, *Avian Pathology* 37(4), 415–220. 10.1080/03079450802216637 18622859

[CIT0013] Lauerman L.H., Hoerr F.J., Sharpton A.R., Shah S.M. & Van Santen V.L., 1993, ‘Development and application of a polymerase chain reaction assay for *Mycoplasma synoviae*’, *Avian Diseases* 37(3), 829–834. 10.2307/1592037 7504919

[CIT0014] Lockaby S.B., Hoerr F.J., Lauerman L.H. & Kleven S.H., 1998, ‘Pathogenicity of *Mycoplasma synoviae* in broiler chickens’, *Veterinary Pathology* 35(3), 178–190. 10.1177/030098589803500303 9598581

[CIT0015] Marois C., Picault J.P., Kobisch M. & Kempf I., 2005, ‘Experimental evidence of indirect transmission of *Mycoplasma synoviae*’, *Veterinary Research* 36(5–6), 759–769. 10.1051/vetres:2005031 16120251

[CIT0016] Moreira F.A., Cardoso L. & Coelho A.C., 2017, ‘*Mycoplasma synoviae* and *Reovirus*: (Re)Emerging infectious diseases in broiler breeders’, *Journal of the Hellenic Veterinary Medical Society* 68(2), 113–122. 10.12681/jhvms.15595

[CIT0017] Nääs I.A., Mollo Neto M., Canuto S.A., Waker R., Oliveira D.R.M.S. & Vendrametto O., 2015, ‘Brazilian chicken meat production chain: A 10-year overview’, *Revista Brasileira de Ciencia Avicola* 17(1), 87–94. 10.1590/1516-635x170187-94

[CIT0018] Nham E.G., Pearl D.L., Slavic D., Ouckama R., Ojkic D. & Guerin M.T., 2017, ‘Flock-level prevalence, geographical distribution, and seasonal variation of avian reovirus among broiler flocks in Ontario’, *Canadian Veterinary Journal* 58(8), 828–834. 28761188 PMC5508928

[CIT0019] Ni Y. & Kemp M., 1995, ‘A comparative study of avian reovirus pathogenicity: Virus spread and replication and induction lesions’, *Avian Diseases* 39(3), 554–566. 10.2307/1591809 8561741

[CIT0020] Reck C., Menin A., Canever M.F. & Miletti L.C., 2013, ‘Rapid detection of *Mycoplasma synoviae* and avian reovirus in clinical samples of poultry using multiplex PCR’, *Avian Diseases* 57(2), 220–224. 10.1637/10425-101712-Reg.1 24689177

[CIT0021] Reck C., Menin A., Pilati C. & Miletti L.C., 2012, ‘Clinical and histologic lesions of mixed infection with *Avian orthoreovirus* and *Mycoplasma synoviae* in broilers’, *Pesqusa Veterinária Brasileira* 32(8), 687–691. 10.1590/S0100-736X2012000800001

[CIT0022] Senties-Cue G., Shivaprasad H.L. & Chin R.P., 2005, ‘Systemic *Mycoplasma synoviae* infection in broiler chickens’, *Avian Pathology* 34(2), 137–142. 10.1080/03079450500058646 16191695

[CIT0023] Sun S.K., Lin X., Chen F., Wang D.A., Lu J.P., Qin J.P. et al., 2017, ‘Epidemiological investigation of *Mycoplasma synoviae* in native chicken breeds in China’, *BMC Veterinary Research* 13(1), 115 10.1186/s12917-017-1029-0 28441945 PMC5405555

[CIT0024] Triant D.A. & Whitehead A., 2009, ‘Simultaneous extraction of high-quality RNA and DNA from small tissue samples’, *Journal of Heredity* 100(2), 246–250. 10.1093/jhered/esn083 18840898

[CIT0025] Xie Z., Fadl A.A., Girshick T. & Khan M.I., 1997, ‘Amplification of avian reovirus RNA using the reverse transcriptase-polymerase chain reaction’, *Avian Diseases* 41(3), 654–660. 10.2307/1592157 9356712

[CIT0026] Zhong L., Gao L., Liu Y., Li K., Wang M., Qi X. et al., 2016, ‘Genetic and pathogenic characterization of 11 avian reovirus isolates from northern China suggests continued evolution of virulence’, *Science Reports* 6, Art ID 35271. 10.1038/srep35271 PMC5067505 27752067

